# CoreView imaging on needle: Rapid core-needle biopsy imaging for point-of-care breast cancer diagnosis

**DOI:** 10.36922/gtm025170039

**Published:** 2025-09-10

**Authors:** Jocelyn R. Jensen, Duy Do, Yuan-ping Chang, Sophia Anderson, Matthew D. Carson, Suzanne Dintzis, Richard M. Levenson, Eric J. Seibel

**Affiliations:** 1 Department of Bioengineering, College of Engineering and UW Medicine, University of Washington, Seattle, Washington, United States of America; 2 Human Photonics Laboratory, College of Engineering, University of Washington, Seattle, Washington, United States of America; 3 Department of Mechanical Engineering, College of Engineering, University of Washington, Seattle, Washington, United States of America; 4 Department of Laboratory Medicine and Pathology, School of Medicine, University of Washington, Seattle, Washington, United States of America; 5 Department of Pathology and Laboratory Medicine, UC Davis Health, University of California, Davis, California, United States of America

**Keywords:** Microscopy with ultraviolet surface excitation imaging, Point-of-care, Global health, Breast cancer, Diagnostics

## Abstract

Breast cancer (BC) is one of the most prevalent malignancies worldwide, with early and rapid diagnosis playing a critical role in improving patient outcomes. Core-needle biopsies (CNBs) are the current gold standard for minimally invasive BC diagnoses. However, in low-resource and rural settings, access to CNB diagnostics is limited by infrastructural constraints, long histopathology turnaround times, as well as financial and geographical barriers. To address these challenges, we developed the CoreView imaging on needle (ION), an affordable, integrated imaging system designed to provide rapid, and point-of-care diagnostic assessment of CNB samples. The CoreView ION integrates microscopy with ultraviolet surface excitation technology, enabling the imaging of tissue biopsy surfaces within 5 min, significantly reducing diagnostic delays. This study presents the design, fabrication, and verification of the CoreView ION prototype operation, including its imaging workflow, staining protocols, and tissue compression testing. Our results demonstrate that the system can successfully generate histology-grade images of porcine and murine fresh biopsies, preserving cellular and nuclear detail of normal and tumor tissue. By streamlining CNB imaging and incorporating mainly manual low-cost components, the CoreView ION has the potential to improve BC diagnostics in low-resource settings, ultimately enhancing early detection and patient care.

## 1. Introduction

Breast cancer (BC) is characterized by dysregulated cell proliferation within breast epithelium, with common types being invasive ductal or invasive lobular carcinoma. Globally, BC affects 2.8 million individuals annually, causing approximately 690,000 deaths.^1,2^ Early diagnosis is crucial for improved patient outcomes, as it significantly impacts an individual’s quality of life and ability to combat the disease. Despite recent advances in preventive treatment measures, BC remains highly prevalent, with one in eight women likely to develop the disease in their lifetime in the United States alone, making it a major focus for treatment improvement.^3^ In low-resource rural settings and low- and middle-income countries (LMICs), BC care presents various challenges and has a much worse 5-year survival rate compared to higher-income countries (HIC).^4,5^ In the United States, for example, the 5-year survival rate is 83.9%; however, in LMICs, such as Gambia, it is as low as 12%.^6^ Overall, 58% of BC deaths occur in LMICs, underlining the critical need for improved diagnostic and treatment methods.^6^ In Ghana specifically, BC is the most common cause of cancer death for Ghanaian women.^7^ The main reason is the late presentation of patients and diagnostic delays.^3–7^ With the introduction of new diagnostic devices, differences in patient care globally can be minimized.

### 1.1. Current clinical practice in BC diagnosis

In current clinical practice, two primary methods are commonly used for minimally invasive tissue sampling: fine-needle aspiration (FNA) and core-needle biopsy (CNB).^8^ While both techniques involve the extraction of cellular material using a needle, they differ in procedure, diagnostic efficacy, and clinical utility. FNA samples utilize smaller needle gauges (22–25 gauge); however, they constitute isolated cells and cell clumps without tissue architecture and thus can be suboptimal for diagnostics, requiring the expertise of a trained cytopathologist to ensure accurate analysis. In contrast, CNB utilizes a larger gauge needle (14–20 gauge) equipped with a spring-loaded cutting mechanism to excise tissue samples from suspected tumors, providing superior diagnostic accuracy, specificity, and sensitivity.^9^

**Figure 1.**
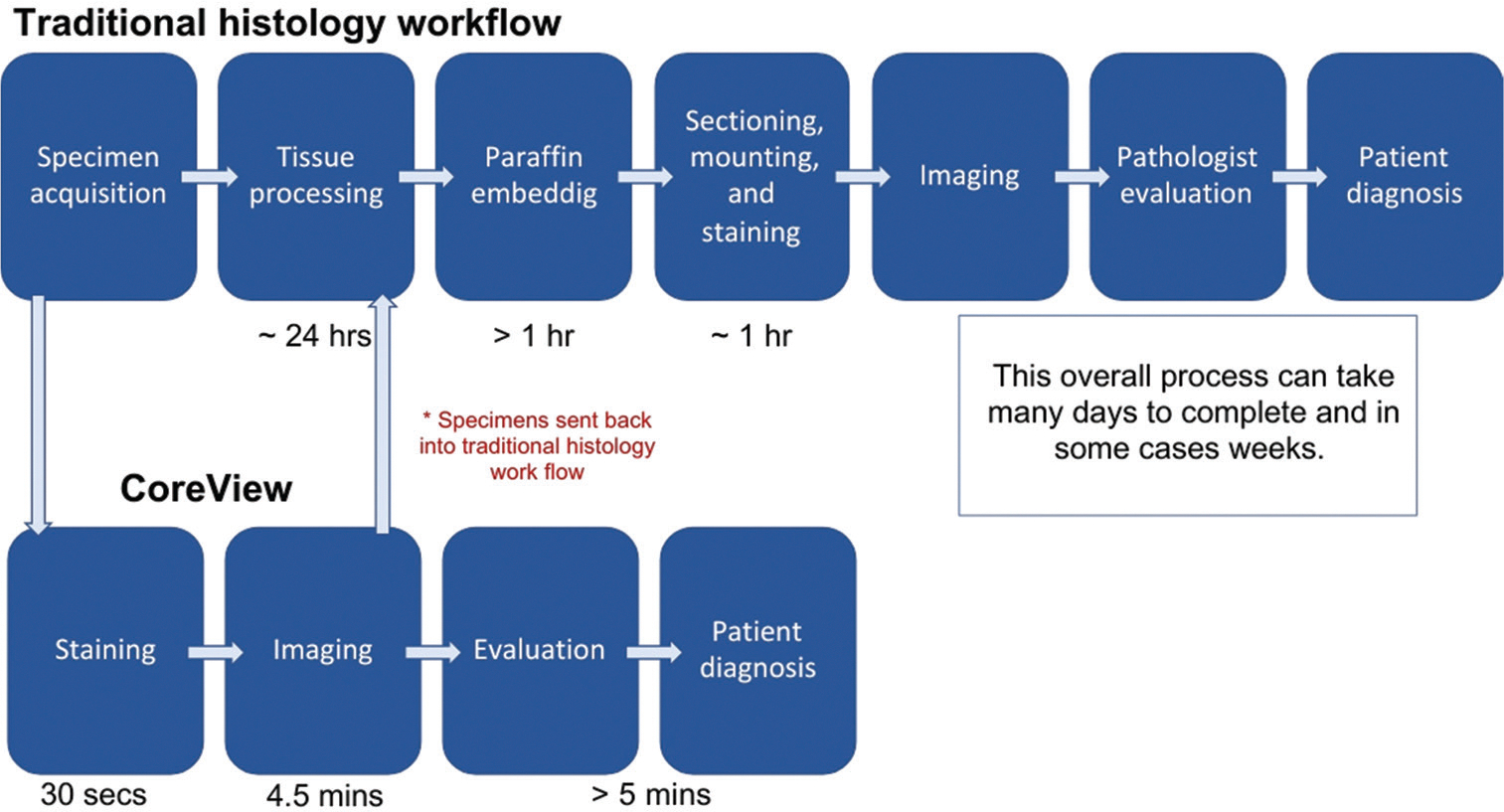
Standard histopathological workflow for biopsy processing. The multi-step procedure includes tissue extraction, formalin fixation, paraffin embedding, sectioning, staining, and pathologist evaluation.

The larger and more structurally intact tissue samples obtained through CNB facilitate histopathological evaluation, making it the standard of care for BC diagnostics.^9^ Despite their diagnostic advantages, CNBs are associated with higher procedural costs and require time-intensive histopathological tissue processing workflows that contribute to delays in BC diagnosis and treatment (Figure 1). In the United States, current trends involve pathologists seeking to optimize the biopsy process by reducing the number of samples required for an accurate diagnosis. Historically, patients underwent 5–10 CNBs per procedure; however, recent studies have indicated that 3–5 cores are adequate for diagnostic or clinical management, and even as few as 2 cores may reliably allow for diagnosis of a malignancy.^10^

### 1.2. Challenges in BC diagnosis in low-resource settings

BC remains a major public health challenge in LMICs, where resource limitations significantly impact diagnosis and treatment. A major constraint is the shortage of essential equipment, inadequate organizational infrastructure, and an insufficient number of qualified personnel within pathology and lab medicine (PALM) services in such areas. PALM services are crucial for accurate disease detection and prognosis; without such services, patients are often uninformed for extended periods without a definitive diagnosis.^11^ Similar challenges are also observed in remote and rural areas of HICs from a lack of funding and continual closing of rural hospitals, restricting access to PALM services.^11,12^

Although CNB plays a critical role in BC diagnosis and treatment planning, the clinical procedure is often constrained by barriers, such as a shortage of trained professionals to precisely acquire cores from the targeted mass, as well as the equipment and supplies required to process the specimens in adequately equipped histology facilities.^13^ More sophisticated techniques, including ultrasound imaging and vacuum-assisted breast biopsy techniques, are not widely implemented due to cost considerations.^14^ Logistical constraints also hinder histopathological processing in LMICs. In low-resource settings, formalin-fixed paraffin-embedded (FFPE) tissue processing and pathologist diagnosis can take up to 3 months to complete, compared to approximately 1 week in high-resource settings.^15^ In Ghana’s eastern region, the lack of local pathologists exacerbates delays, as samples must be sent off-site for evaluation.^16^

Early BC detection is further limited by healthcare barriers and social stigma, resulting in many patients only presenting with advanced disease in clinics. Studies indicate that 20–30% of women with BC symptoms delay seeking medical care for at least 3 months.^3,17,18^ In addition to limited healthcare infrastructure, the medical cost associated with BC diagnosis and treatment is a barrier for many, and prevents timely diagnosis and treatment.^19^

### 1.3. The CoreView imaging on needle (ION) project

In Ghana, treating cancer involves many indirect expenses that are not limited to those incurred during treatment. A recent survey conducted among individuals seeking cancer care in Ghana revealed that only 54.8% of the costs were solely medical, whereas direct non-medical and indirect costs from seeking treatment made up 7.1% and 38.1% of the overall expenses.^20^ Such costs included the transportation fees, caregiver fees, and the loss of productivity, deterring patients from seeking necessary follow-up care. As a result, a significant portion of individuals undergoing CNB never receive a definitive diagnosis after biopsy, further hindering effective BC management.^21^ One way to mitigate the indirect cost barriers is to introduce lower-cost portable laboratory equipment into rural areas and rural healthcare clinics.

The Human Photonics Laboratory at the University of Washington (UW) has aimed to create a low-cost, portable device that requires minimal electrical needs and training to operate, allowing for rapid, point-of-care diagnosis during a patient’s first visit in LMICs and other low-resource areas. The CoreView ION is designed as a cost-effective, accessible imaging solution that produces diagnostic-quality results while minimizing the need for specialized training (Video S1). To overcome the challenges associated with traditional CNB histopathology, the CoreView ION implements manual low-cost components to simplify operation and maintenance, as well as training protocols. By significantly reducing diagnostic turnaround time, this approach has the potential to improve patient outcomes and decrease the number of women in underserved regions who remain undiagnosed due to a multitude of barriers stacked against them.

## 2. Materials and methods

### 2.1. Design and fabrication of the prototype of CoreView ION

**Figure 2.**
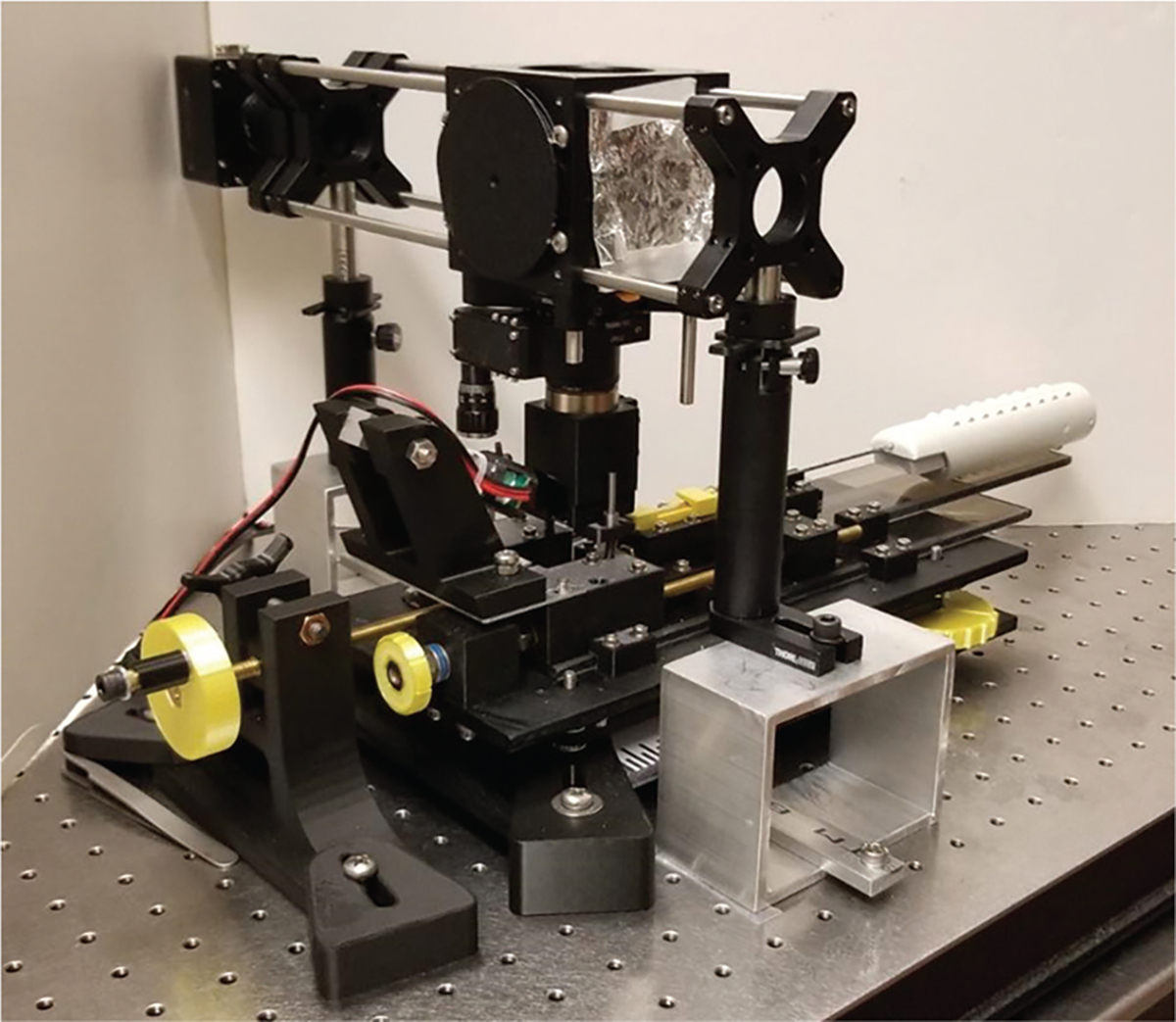
The CoreView imaging on needle prototype. The fixture module, modeled in SolidWorks initially and fabricated using a three-dimensional fused filament fabrication printer, features a structured carbon polycarbonate frame and a custom microscope holder for imaging core needle biopsies while still on the needle. The full prototype was designed to be low-powered, low-cost, and compact, allowing for increased portability.

To facilitate imaging of CNBs while still on the needle, a prototype fixture was designed, iterated, and tested on animal tissues (Figure 2). Prototype components were modeled using SolidWorks (Dassault Systèmes SolidWorks Corp., USA) and fabricated with a three-dimensional (3D) fused filament fabrication (FFF) printer (Prusa Research a.s., Czech Republic). The CoreView fixture consists of a frame made from structured carbon polycarbonate plates attached to a custom microscope holder. This initial prototype utilized CNB preparation on the needle, employing only drops of fluorescence dye and hand-rinsed saline. Furthermore, future prototypes can integrate CNB staining and rinsing in an automated process (Video S2).

The biopsy is acquired using 14–18-gauge needle biopsy guns and then stained with Rhodamine B and Hoechst. Following staining, the needle biopsy gun is loaded onto a 3D-printed holder. A hand crank is turned to position the specimen against the surface of the fixed, UV-transparent window, which is preset to be the focal plane of the objective lens.

The CoreView ION prototype is equipped with Nikon 4× and 10× objective lens imaging under UV low-powered light-emitting diode (LED) illumination, with multi-axis movement control for both needle biopsy and biopsy compression. The imaging workflow involved staining tissue with Rhodamine B (counterstain, 10 mg/mL) and Hoechst fluorescence dye solutions (Hoechst 33342 nuclear stain, 5 mg/mL), loading the CNB onto the microscope stage, compressing the biopsy surface against a clear quartz coverslip window, and capturing images within 5 min using microscopy with ultraviolet surface excitation (MUSE) technology.^22^ Video S2 illustrates the fully automated CoreView ION system, showcasing each integrated component of the final prototype for clear visualization.

### 2.2. Imaging workflow

**Figure 3.**
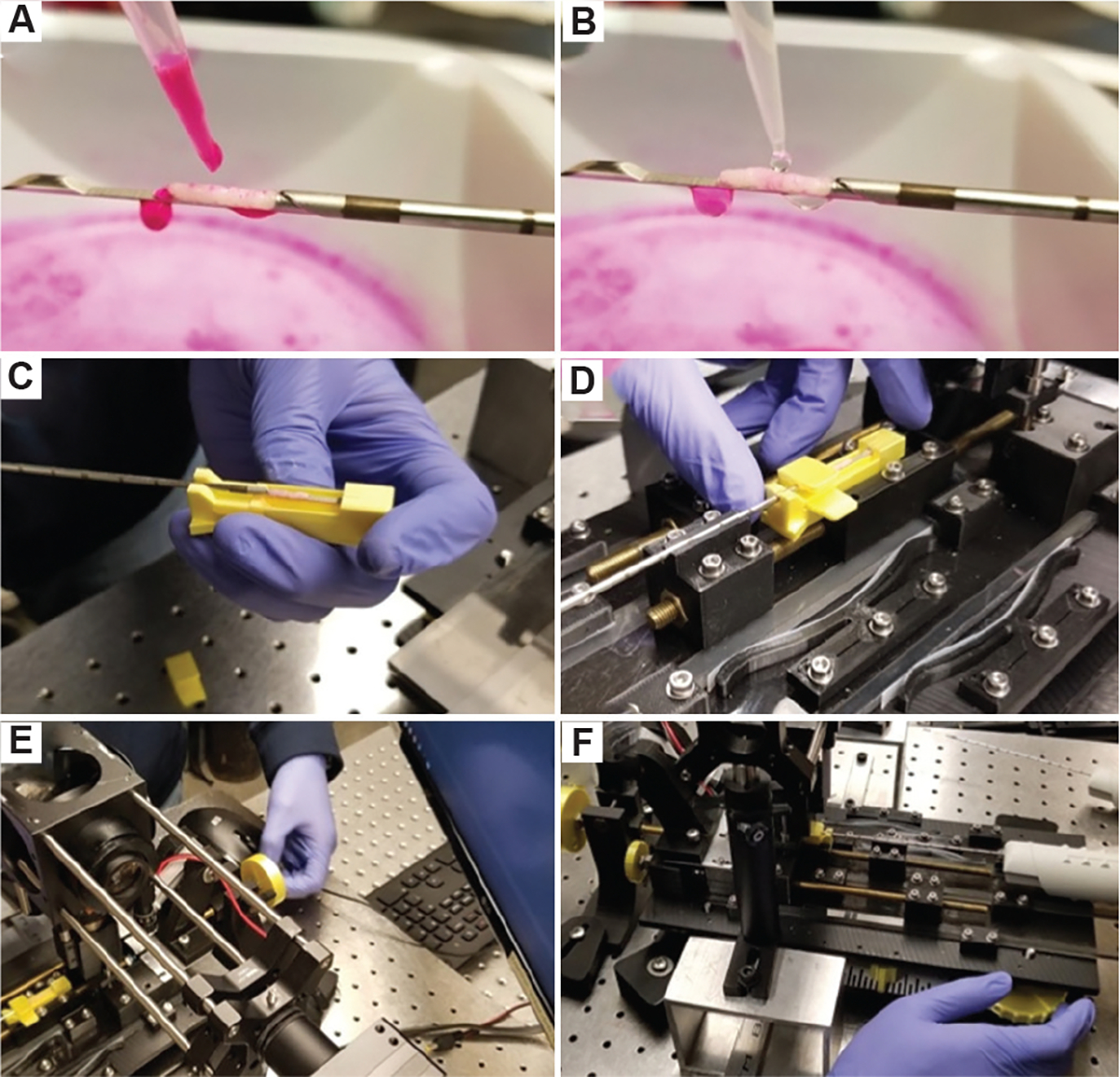
CoreView imaging on needle workflow. (A) After the biopsy is acquired, Rhodamine B and Hoechst staining solutions are applied onto the sample, (B) the sample is then rinsed with PBS solution, (C) The biopsy gun is loaded into a three-dimensional-printed holder, (D) the holder is locked into the fixture, (E) a hand crank is used to move the biopsy under the quartz coverslip along the long y-axis, and (F) the biopsy is raised on the z-axis to compress the sample against the coverslip for clear imaging.

The current imaging workflow consists of manual staining and loading (1.5 min), MUSE fluorescence imaging while axially scanning the CNB (3 min), and unloading the biopsy (0.5 min), resulting in a total processing time of 5 min from biopsy collection to diagnostic image acquisition (Figure 3). The removal of the CNB from the needle into buffered 10% formalin for conventional downstream processing is the only time the tissue is handled after the core acquisition, which allows for a more pristine surface for MUSE imaging. The MUSE imaging has been shown not to affect conventional hematoxylin and eosin (H&E) imaging of the thin sections taken from the conventional FFPE processing of the CNB.^22^

CNBs were obtained from tissue using a 14-gauge tissue biopsy needle (MC1416 MaxCore, Becton Dickinson/Bard, USA). Following the biopsy procedure, tissues were rinsed with PBS to remove excess debris. A Hoechst and Rhodamine B staining solution was applied until the biopsy top surface was fully wetted. After 30 s, the biopsy was rinsed with PBS to prevent overstaining (Figure 3B). The biopsy needle was then secured in a 3D-printed holder for stability and positioned within the CoreView demonstrator (Figure 3C and D). A hand crank on the left-most end of the demonstrator was used to align the CNB for imaging (Figure 3E). Once aligned, the CNB was brought into contact with a fixed UV-transparent imaging window by adjusting a hand crank, ensuring optimal imaging conditions of a partially flattened CNB surface (Figure 3F).^23^ Overhead white lights were turned off, and UV illumination was applied. The images were captured using the Ximea imaging application (XIMEA GmbH, Germany) and Ximea camera (xiD MD091CU-SY, XIMEA GmbH, Germany).

MUSE imaging was performed using 280 nm UV LED light for fluorescence excitation. The Hoechst stain is selectively bound to nuclear material, while Rhodamine B counterstains cytoplasm and surrounding stroma, as well as other structures. Two different objective lenses were utilized for imaging tissue samples. With the 4× objective lens, each biopsy required approximately 10 images to encompass the entire specimen. Images were acquired with an exposure time of 10 s and a 10 dB gain, using 20% overlap for subsequent stitching. With the 10× objective lens (numerical aperture = 0.3), each biopsy required approximately 25 images before stitching, using the same imaging parameters as the 4× objective lens. Images were stitched using ImageJ software (National Institutes of Health, USA).

### 2.3. Compression testing for biopsy integrity

To determine the extent of compression that can be applied to breast CNBs while preserving tissue integrity for downstream histopathological analysis, compression testing was conducted using both *ex vivo* porcine tissue and a murine tumor model. Fresh CNBs were obtained from *ex vivo* porcine tissue, and the murine tumor FVB/N-Tg (TgMMTV-neu) mouse strain was used as a representative model for human mammary tumors obtained from the Cancer Vaccine Institute in Seattle, Washington, using 14-gauge needles. A total of approximately 20 samples were analyzed for each tissue type across different compression levels.

**Figure 4.**
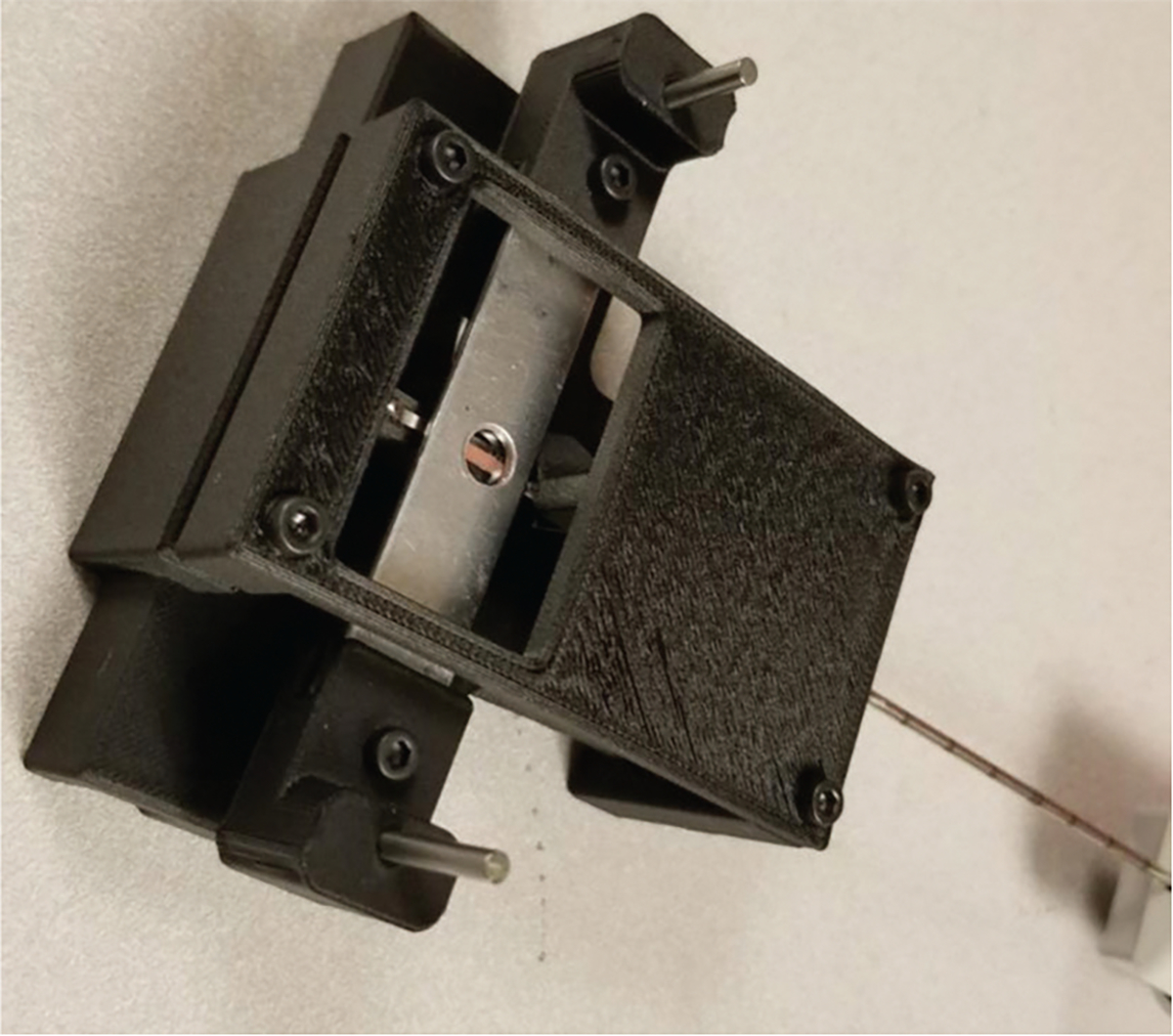
Compression testing device. This simple device enables quantitative assessment of tissue deformation while maintaining histopathological integrity.

For porcine tissue, biopsy thickness was measured on the biopsy gun using a caliper before compression. To prevent tissue dehydration, biopsies were showered with PBS solution before being compressed. A screw-based glass-slide compression device was used (Figure 4), consisting of two 3D-printed round disks, each marked with 16 evenly spaced reference points corresponding to a 0.03215 mm increment of compression. The disks were attached to two M3 hex socket screws with a 0.5 mm pitch, ensuring uniform compression across the porcine biopsy specimen. Biopsies from fresh pig breast tissue were compressed to 50%, 40%, and 30% of their original thickness, and calculated using Equation I.


(I) $$\#\;\text{Marks}=\frac{\begin{array}{ll} \begin{array}{l} \text{Original}\\ \text{thickness} \end{array} & -\left(\begin{array}{l} \text{Original}\;\text{thickness}\\ \;\times \;\text{\%}\;\text{compression} \end{array}\right) \end{array}}{0.03125\;\text{mm}}$$

For instance, a 1.2 mm thick biopsy required approximately 23 marks of screw rotation to achieve 40% compression. Table 1 shows this method and the corresponding thicknesses. Two biopsies were collected for each compression condition, and compressed biopsies remained under applied pressure for 2 min before fixation in 10% neutral-buffered formalin. As controls, two additional biopsies were left uncompressed for 2 min before fixation in 10% neutral-buffered formalin for 72 h.

A calibrated scale was integrated into the CoreView fixture for murine biopsies, enhancing precision in determining biopsy compression levels. This scale was designed based on the average height of a 14-gauge CNB (1.2 mm) and featured black notches spaced at 50 μm increments. In the murine model, biopsies were compressed to 70%, 60%, 50%, and 40% of their original thickness. Before compression, these biopsies were stained with Hoechst and Rhodamine B solutions for 30 s, followed by rinsing with PBS. CNBs were then loaded onto the CoreView demonstrator for controlled compression and imaging using the MUSE microscope. Following imaging, biopsies were fixed in 10% neutral-buffered formalin for 72 h before submission for histological processing.

Following fixation, all specimens were submitted to the UW Histology and Imaging Core for routine H&E staining and imaging. A blinded histopathological evaluation was conducted by a breast pathology specialist, who assessed image sets corresponding to the control and compressed conditions. Each set was evaluated for diagnostic quality and the presence of compression-induced artifacts to determine the effects of controlled compression on biopsy integrity.

### 2.4. Quantification of nuclear edge sharpness using ImageJ

To quantify nuclear edge sharpness, grayscale 10× images of porcine tissue sections imaged using MUSE and conventional H&E brightfield images were analyzed in ImageJ. The scale was set using known reference length of full porcine biopsies (~1 cm in length) and used to calibrate the image scale, spanning a distance of 10,000 μm across 38,702 pixels. Using the Plot Profile tool, intensity values were measured across the diameter of five representative nuclei per imaging modality. For each profile, the minimum and maximum grayscale intensities were recorded, and the 20% and 80% intensity levels from baseline were calculated. The pixel distance between these two points was used as a quantitative measure of how sharply intensity changed at the nuclear boundary. Average distances were computed for each modality to compare edge gradients between MUSE and H&E images.

## 3. Results and discussion

### 3.1. MUSE imaging of porcine tissues

**Figure 5.**
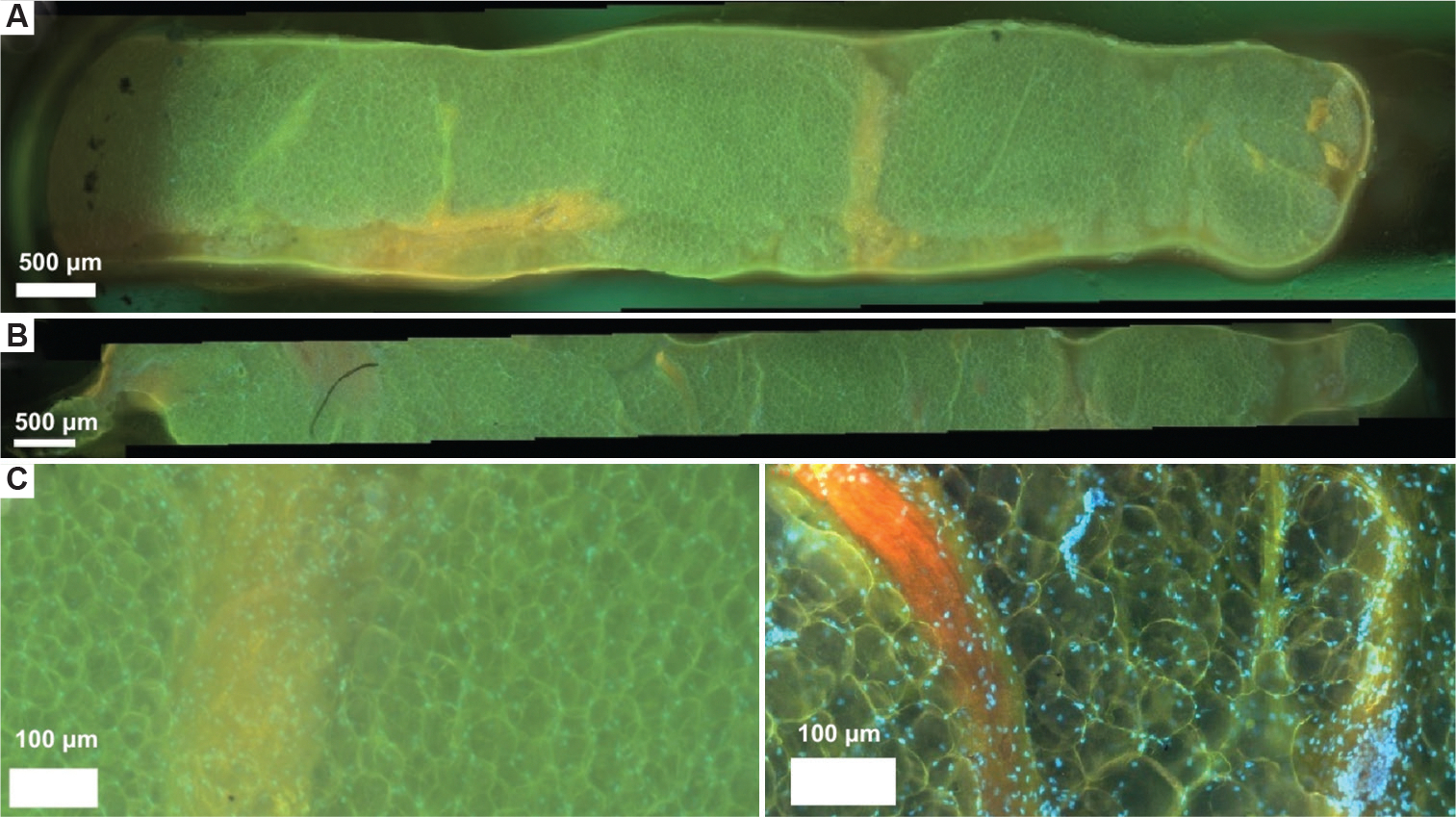
Microscopy with ultraviolet surface excitation imaging of fresh porcine breast tissue obtained via 14-gauge core needle biopsy gun. (A) Pig breast sample imaged using a 4× objective lens, stitched with ImageJ. Scale bar: 500 μm; magnification: 10×, (B) Pig breast sample imaged using a 10× objective lens, stitched with ImageJ. Nuclei are stained with Hoechst and appear blue/teal compared to the Rhodamine B counterstain. Scale bar: 500 μm; magnification: 10x, (C) Zoomed-in 4× MUSE image of pig breast tissue as seen in (A). Scale bar: 100 μm; magnification: 4×, (D) Zoomed-in 10× MUSE image of pig breast tissue as seen in (B). Scale bar: 100 μm; magnification: 4×.

Using the CoreView prototype, we successfully imaged fresh pig breast tissue within 5 min after biopsy acquisition, well within the 1–2 h post-ischemic time target for tissue specimens before formalin fixation. The rapid imaging workflow demonstrated the potential for near-real-time evaluation of tissue morphology, a crucial factor in point-of-care applications. The resulting panoramic images exhibited preservation of cellular architecture, with well-defined nuclear contrast and strong contrast between nuclei and the surrounding stromal components (Figure 5). While these findings are promising, it is important to note that no human tissues were used in this study; further validation with human biopsy samples will be necessary to assess clinical applicability and ensure translational relevance.

Despite the clarity of nuclear features, challenges were observed in capturing detailed imaging of ductal structures within the pig breast tissues. This limitation may be attributed to differences in glandular composition between porcine and human breast tissue, to variations in tissue density and properties that influence optical penetration and contrast, and to the ability of the needle biopsy gun to sample targeted areas.

### 3.2. MUSE imaging of murine tumor models

**Figure 6.**
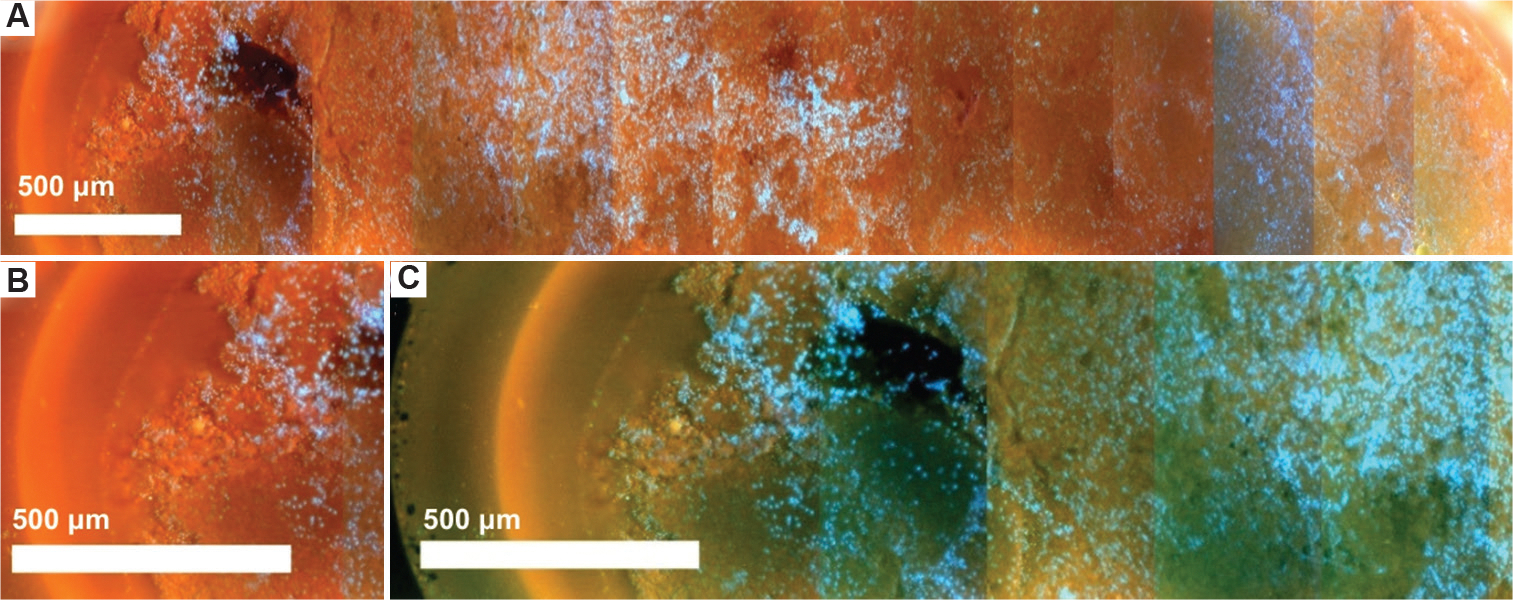
Microscopy with ultraviolet surface excitation imaging of a mouse tumor model obtained via a 14-gauge core needle biopsy gun. (A) Image of the mouse tumor, stitched using ImageJ. Scale bar: 500 μm; magnification: 10×, (B) Zoomed-in segment of the tumor from (A), stitched using ImageJ. Nuclei are stained with Hoechst with a Rhodamine B counterstain. Scale bar: 500 μm; magnification: 10×, (C) Color-adjusted image using ImageJ to improve visual comparison between pig breast biopsies and mouse tumor biopsies. Scale bar: 500 μm; magnification: 10×.

Mouse tumor samples from the FVB/N-Tg (TgMMTV-neu) mouse strain, provided by the Cancer Vaccine Institute in Seattle, WA, were utilized as additional specimens. The tumor images were captured using the CoreView prototype after biopsy acquisition and staining (Figure 6).

The resulting images demonstrated the system’s capability to visualize cancerous specimens, which exhibited distinctly different density and tissue properties compared to the pig breast tissues previously tested. While the images provided valuable feedback on the device’s ability to assess diseased tissue, overall image clarity was lower than that observed in pig breast tissue. Notably, nuclear features in the murine samples appeared with limited structural detail, and overall tissue architecture was poorly defined. Several factors may contribute to the reduced image clarity observed in the murine tumor sample. First, inconsistencies in later quartz coverslip cleaning likely introduced optical artifacts, such as blurring. In addition, as these tumors were obtained as residual specimens, the tissue had been acquired a considerable period before imaging and had experienced 6 h of ischemic time, resulting in tissue degradation and loss of structural integrity. Furthermore, the staining protocol using Rhodamine B and Hoechst may have influenced the image brightness and contrast, potentially obscuring finer morphological details. Future studies will aim to refine tissue preparation protocols and optimize staining conditions to improve imaging quality and consistency across different tissue types.

**Figure 7.**
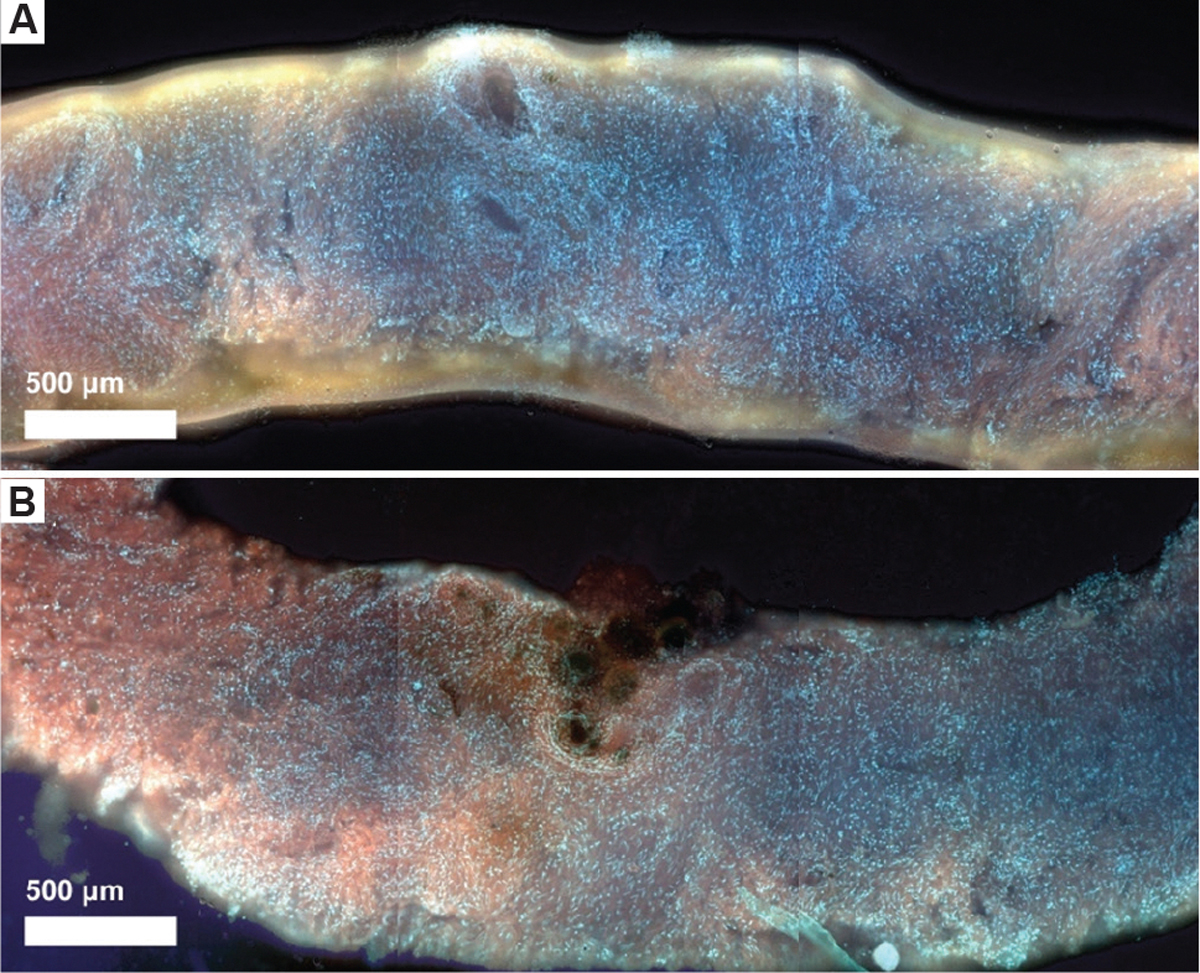
High-quality MUSE images from core biopsies in non-needle-based applications. (A) Fresh normal prostate core biopsy stained with alternative Rhodamine and Hoechst, imaged using MUSE by the Levenson Lab, University of California, Davis. Scale bar: 500 μm; magnification: 10×, (B) Fresh cancerous prostate core biopsy stained with alternative Rhodamine and Hoechst, also imaged using MUSE by the Levenson Lab, University of California, Davis.^22^ Scale bar: 500 μm; magnification: 10×. Abbreviation: MUSE: Microscopy with ultraviolet surface excitation.

Despite the suboptimal results observed in the murine tumor samples, high-quality MUSE images have been successfully obtained from core biopsies in non-needle-based applications (Figure 7A and B). These comparative images demonstrate the level of image clarity achievable with optimized sample handling and preparation, further supporting the notion that, with continued refinement, the CoreView ION platform holds strong promise for rapid, point-of-care diagnostic applications.

### 3.3. Biopsy integrity under compression testing

Understanding the effects of compression on biopsy samples is critical since the prototype requires a flat surface for planar microscopic imaging. When the biopsy is pressed against the imaging window, the specimen is flattened, allowing for a greater percentage of the tissue surface in full focus and direct contact with the quartz coverslip, improving image quality and resolution. However, CNBs are structurally fragile, and external stressors can lead to mechanical breakdown. Therefore, confirming if compression leads to tissue damage that could compromise downstream histopathological analysis is essential.

**Figure 8.**
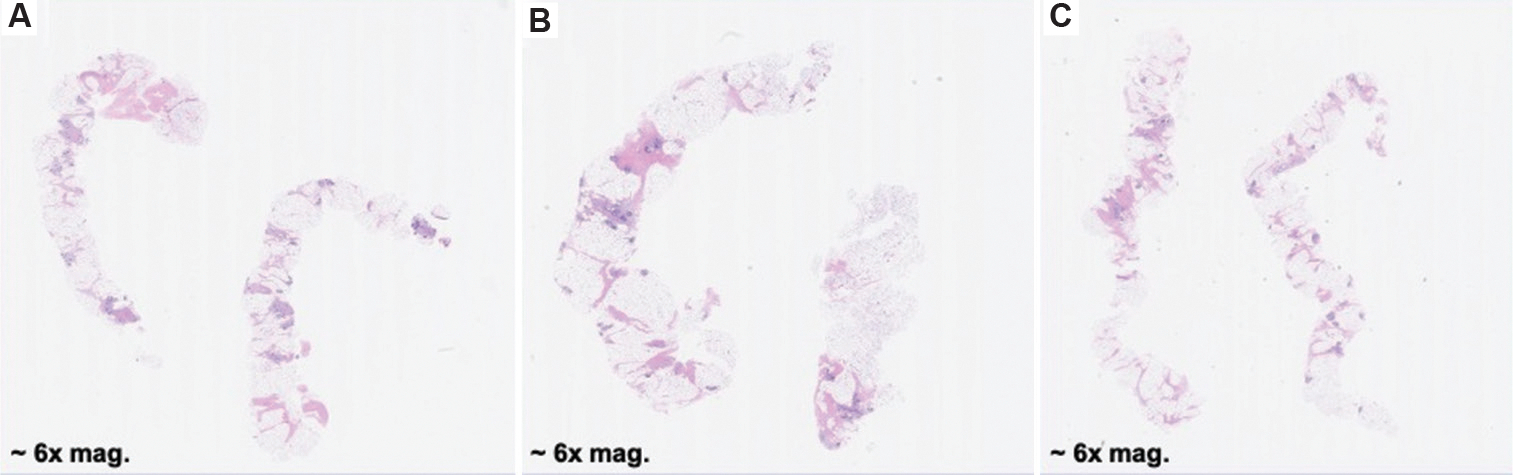
H&E scans of pig breast biopsies. (A) H&E slide of non-diseased porcine breast biopsy compressed to 50% of original thickness, showing no compression artifacts. Magnification: 6×, (B) H&E slide of non-diseased porcine breast biopsy compressed to 40% of original thickness, showing no compression artifacts. Magnification: 6×, (C) H&E slide of non-diseased porcine breast biopsy compressed to 30% of original thickness, showing no compression artifacts. Magnification: 6×. Abbreviation: H&E: Hematoxylin and eosin.

After conducting a study to assess the effects of varying compression levels on biopsy integrity, the pig breast samples were processed through a standard histopathological workflow at the UW Histology and Imaging Core for H&E staining (Figure 8). The resulting digital pathology slides were reviewed by a breast pathology specialist from the UW Medicine Department of Pathology. On evaluation, the pathologist remarked, “The tissue quality and staining are excellent… able to make a diagnosis on tissue samples of this quality” (Dr. Suzanne Dintzis, MD, PhD, October 28, 2022). These findings, later confirmed by a board-certified pathologist from the University of California, Davis, Department of Pathology, indicate that even under high compression—flattening the specimen to 30% of its original thickness, more than the compression applied using the CoreView ION system—the tissue maintained structural and molecular integrity. This preservation suggests that the samples will remain viable for downstream pathological assessment.

It is important to note that while the compression mechanism utilized in this study differs from that of CoreView ION, the employed test fixture allowed for more precise compression control, as well as improved specimen accessibility and processing speed. One limitation of this study is the absence of cancerous tissue in the test samples; while healthy tissue demonstrated no observable differences across compression levels, diseased tissue may respond differently. Further studies incorporating malignant samples are necessary to evaluate potential compression-induced artifacts.

**Figure 9.**
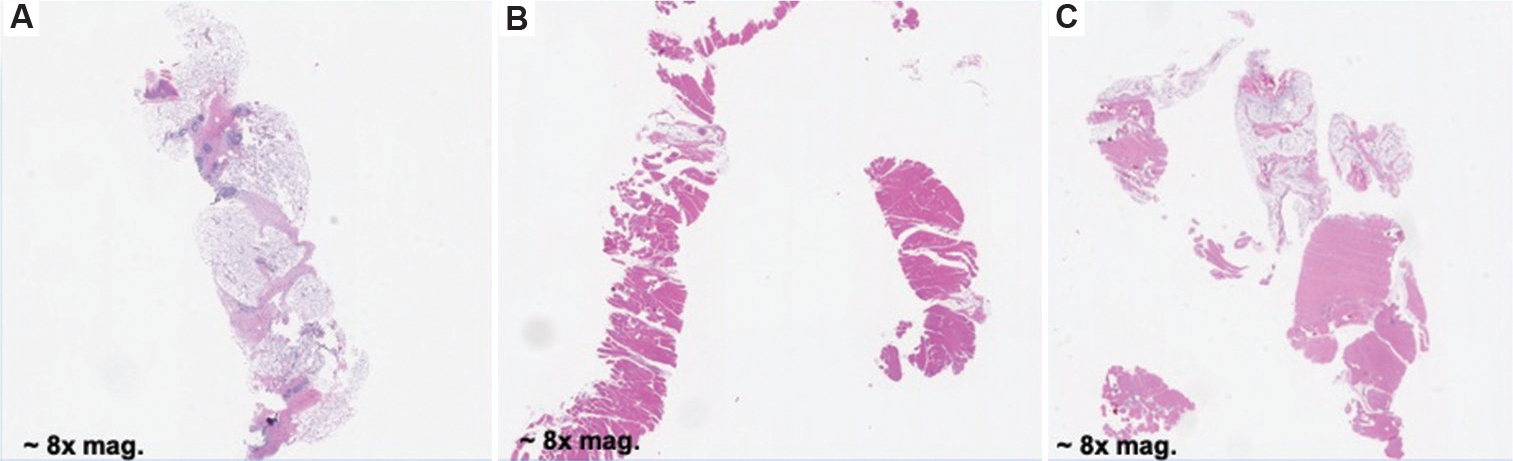
H&E scans of mouse tumor biopsies. (A) Murine tissue compressed to 70% of its original thickness. Magnification: 8×, (B) Murine tissue compressed to 60% of its original thickness. Tissue artifacts occurred during histology processing, resulting in a fragmented sample. Magnification: 8×, (C) Murine tissue compressed to 50% of its original thickness. Tissue artifacts occurred during histology processing, resulting in a fragmented sample. Magnification: 8×. Abbreviation: H&E: Hematoxylin and eosin.

Murine tissue sections were also submitted to the UW HIC for H&E staining and subsequent pathological evaluation (Figure 9). The digital slides were reviewed by a breast pathologist, who similarly observed that compression did not appear to compromise image integrity or impede accurate diagnosis. However, these samples consisted of spontaneous mammary tumors in mice, necessitating additional validation using human breast tissue to rule out the possibility of compression-induced artifacts. Murine pathology differs significantly from human pathology due to inherent structural variations, including a greater density of hair follicles, differences in stromal composition, and variation in glandular architecture. These distinctions underscore the importance of follow-up studies in human tissue to ensure the translatability of findings to clinical practice.

### 3.4. CoreView ION prototype performance

**Figure 10.**
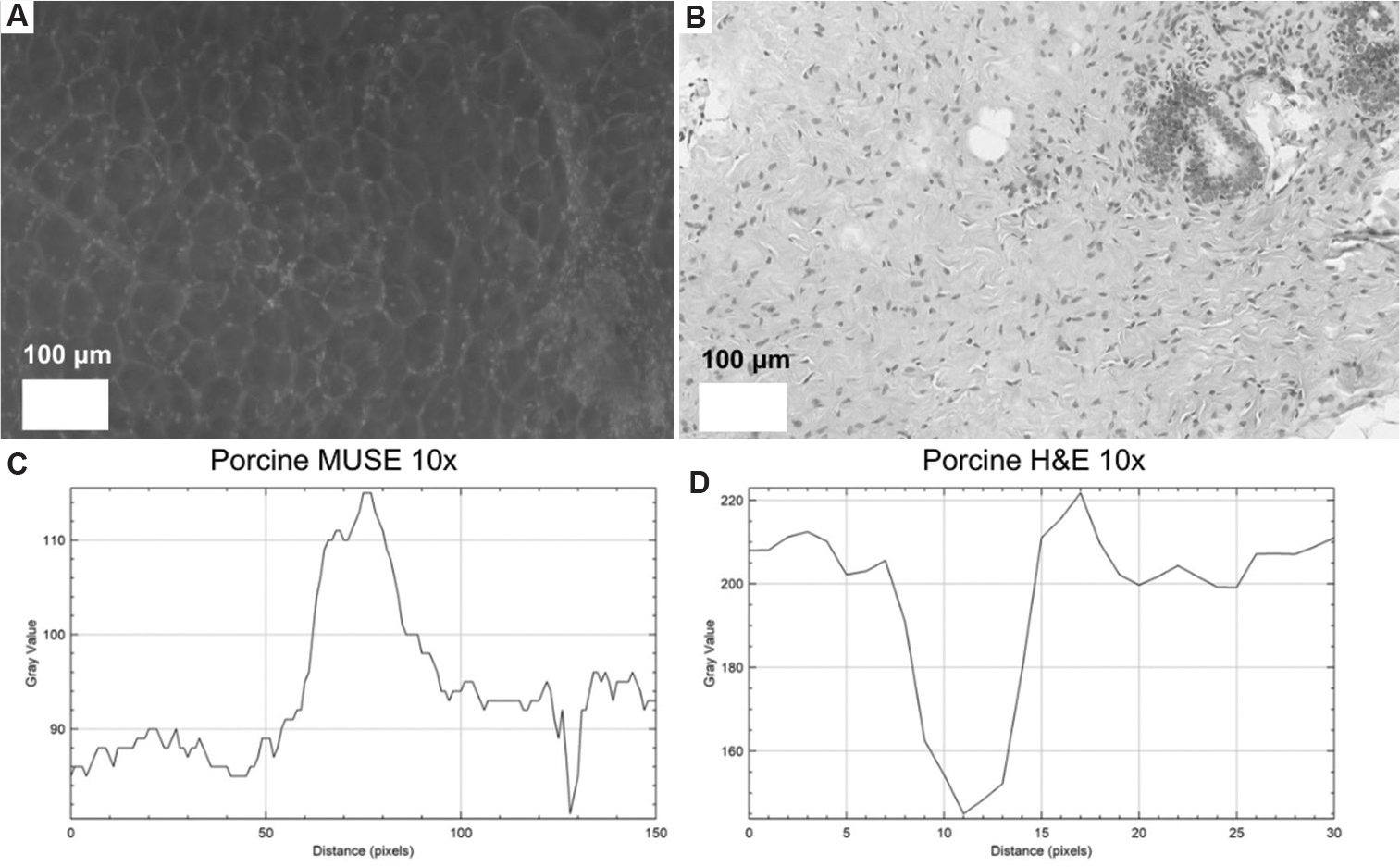
Grayscale MUSE and H&E porcine images with resolution analysis. (A) Grayscale porcine MUSE image. Scale bar: 100 μm; magnification: 10×, (B) Grayscale porcine H&E image, shown for resolution comparison. Scale bar: 100 μm; magnification: 10×, (C) Intensity profiles across representative nuclei of (A); (D) Intensity profiles across representative nuclei of (B). Abbreviations: H&E: Hematoxylin and eosin; MUSE: Microscopy with ultraviolet surface excitation.

Grayscale 10× porcine tissue images acquired using MUSE and H&E staining were compared to assess nuclear contrast and edge sharpness. In the MUSE image, nuclei appear brighter than the surrounding stroma, whereas in the H&E image, nuclei are darker. Intensity profiles across representative nuclei demonstrate this inverse contrast pattern, with the MUSE signal increasing in nuclear regions and the H&E signal decreasing due to the dark hematoxylin stain. Relative quantitative analysis showed that the average pixel distances, used as a measure of nuclear edge sharpness, ranged from 20% to 80% of the normalized intensity range, were 10 pixels in MUSE images and 8.2 pixels in H&E images. This indicates that MUSE provides positive nuclear contrast with a gradual transition at nuclear boundaries compared to the steeper edge seen in H&E-stained sections (Figure 10 and Table 2). Notably, our UV dose was approximately 20 times lower than that used in previous MUSE studies. We employed unfocused illumination with longer camera dwell times to reduce light intensity and minimize photobleaching.^24^

The CoreView ION fixture demonstrates the capability to generate diagnostic-quality images within a remarkably short timeframe, producing a complete image within 5 min with no failures. By remaining on the biopsy needle, the biopsy stayed intact until the compression step in the imaging process. When an earlier version of CoreView used a fluidic lab-on-chip approach without compression, the removal process recovered only 90% intact CNBs for fresh breast tissue, necessitating the CoreView ION approach.^25^ This efficiency is a key advantage for rapid diagnostic workflows, allowing for near-instantaneous feedback during pathology assessments and CNB procedures. The prototype’s ability to produce high-quality digital images at low magnification shows its potential as a viable alternative to conventional histopathology techniques (Figure 1). The images maintain diagnostic integrity, enabling pathologists to analyze tissue samples effectively without the need for traditional histological processing steps.

Furthermore, image compression was evaluated to determine its impact on downstream histopathological analysis, including tests with both pig and mouse tissue samples. Results indicated that compression does not compromise the integrity of downstream histopathological analysis, and the samples remain viable for pathological evaluation in a standard workflow. Compression allowed for sharper image quality across the length of the CNB and expanded the area being imaged by up to two times the original area.

A significant strength of the system lies in its minimal requirements for electrical power. The CoreView prototype operates with only three components requiring electrical power (LED: 1.5W, Ximea camera: 3.0W, and a computer, which can be a battery-powered laptop), showing promising proof-of-concept work for low-cost and accessible solutions for rural and low-resource clinical settings. With a total cost of goods of less than USD 8,000 (excluding labor), including a camera costing USD 4,000, the system offers an affordable option compared to existing digital pathology solutions. While the CoreView ION has not been fully automated, this was found unnecessary for achieving rapid imaging and analysis, specifically for the stain protocol. The simplicity and speed of the system suggest that automation could easily be implemented in future iterations, but even in its current form, the workflow remains efficient and practical. If further automation and higher-powered LEDs were implemented, the 5-min process could be even faster while minimizing errors. With increased speed provided by system automation, a 20× objective could be implemented at an incrementally higher cost.

### 3.5. System limitations and challenges

**Figure 11.**
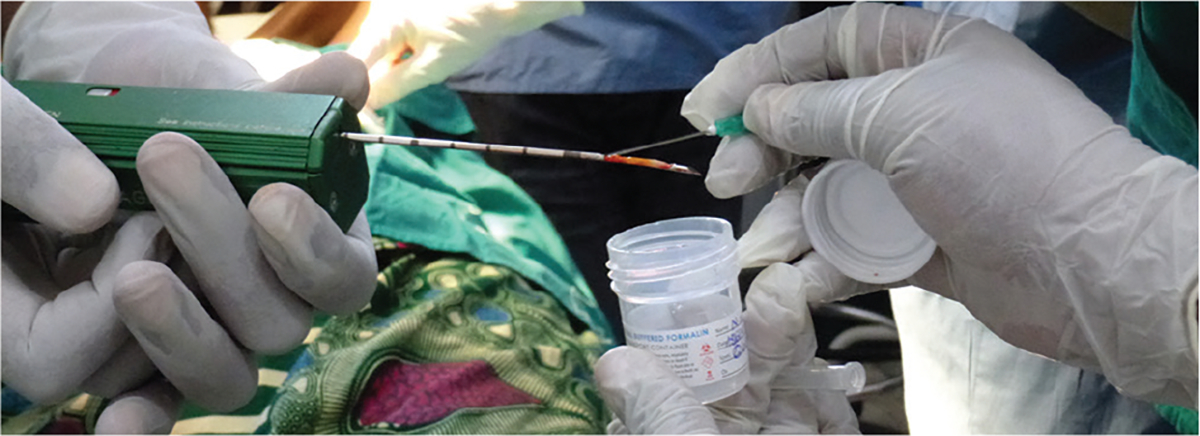
Reusable BARD coring needle handpiece and low-cost disposable needle are displayed to the photographer during a training course in Rwanda on breast biopsy procedure for palpable breast masses. The photograph is provided by Dr. Jane Brock, formerly at Brigham and Women’s Hospital, Harvard University.

While the CoreView prototype offers a promising proof-of-concept for rapid and low-cost imaging of CNBs in BC diagnostics, several limitations must be considered before clinical implementation. One major limitation is the expectation that a single core is sufficient for a diagnosis. If a second core is needed, then the needle would necessitate a thorough cleaning and rinsing protocol, introducing potential workflow inefficiencies and requiring further validation for sterility. An example of global use of a reusable CNB device (Figure 11), which could be incorporated into a CoreView ION imaging workflow, with multiple clean needles being used with one reusable biopsy gun.

Another issue with the system is the dependence on quartz coverslips, which are significantly more expensive than standard glass slides. This cost factor may present a barrier to widespread adoption, particularly in low-resource, rural settings where affordability is a primary concern. Between each sample, the quartz glass required cleaning or replacing if broken, leading to workflow inefficiency between samples.

In addition, the imaging workflow and staining process remain unoptimized. The current staining and imaging parameters were developed as proof-of-concept and have not yet been refined for clinical-grade imaging. Further optimization is necessary to enhance contrast, reduce imaging artifacts, and improve overall diagnostic quality. Furthermore, the study has not yet demonstrated high-quality imaging of malignant BC human tissues. The initial results provide a foundation for future work, but additional validation using a diverse range of cancerous tissues is required to assess the system’s true diagnostic potential. These limitations highlight areas for future improvement, including optimization of the staining and imaging workflow, cost reduction strategies, and expanded validation studies to ensure clinical applicability with and without artificial intelligence (AI) enhanced diagnosis from the resulting CoreView ION images.

### 3.6. Future improvements and optimization

As the CoreView ION is an initial proof-of-concept prototype, there are potential directions for further refinement of the imaging strategy and design. At present, the prototype depends on a computer system for MUSE imaging, necessitating access to electrical power and a computer connection. However, recent studies have demonstrated the feasibility of utilizing MUSE imaging through smartphones.^24^ The Pocket MUSE system, which employs an optical module attached to the rear lens of a smartphone, facilitates high-quality fluorescence imaging at a significantly reduced cost. Incorporating Pocket MUSE technology and concepts into the CoreView ION could eliminate the requirement for a computer connection, enhancing its usability in rural settings. Furthermore, the existing low-powered UV LEDs in the current fixture could potentially be replaced with a battery-powered module, allowing the system to operate solely on battery power.

The current prototype relies on a clean transparent coverslip, which introduces workflow inefficiencies and cleaning challenges. Recent advancements in imaging technologies, such as fluorescence-imitating brightfield imaging (FIBI), have demonstrated the capability to capture tissue images without coverslip compression.^26,27^ Eliminating the need for a clean glass surface would streamline the imaging process. Integrating FIBI thick tissue imaging, which can produce a 30× greater signal-to-noise ratio compared to MUSE images, can accelerate the imaging process and eliminate UV optical hazards, as well as the need for expensive quartz glass. Although the extended depth of focus algorithm would eliminate the need for tissue compression, its deployment requires fast axial scanning, which would increase cost and complexity for the future portable CoreView ION system. While these technological adaptations are promising for low-resource and point-of-care applications, clinical translation will ultimately require validation using human biopsy specimens to confirm clinical diagnostic accuracy and performance. Expanding accessibility and accelerating diagnosis with AI-enhanced analysis of thick tissue biopsy images are within reach, as these pre-clinical results suggest that the CoreView instrument can provide rapid, point-of-care diagnosis for the most prevalent cancers in Africa: BC, cervical cancer, and prostate cancer.^28–32^

## 4. Conclusion

The CoreView ION system demonstrates the feasibility of rapid, on-needle imaging for CNB analysis, offering a potential low-cost point-of-care solution for BC diagnostics. By integrating MUSE imaging with a more streamlined workflow, this system enables bedside visualization of biopsy samples, compared to the days to months of delays experienced now in low-resource settings. Further optimization and clinical validation with human tissue will be necessary to fully establish its role in improving BC survival rates by providing greater access to these new rapid diagnostic pathways. This approach is a significant advancement in pathology, using thick tissue biopsy imaging (MUSE and FIBI), as on-needle imaging has not been previously explored. By enabling real-time evaluation at the point of care, the CoreView ION system has the potential to greatly reduce patient burden, expedite treatment decisions, and ultimately improve clinical outcomes in BC diagnostics.

## Supplementary Material

Supplementary video 1 **Video S1.** Video demonstration of the CoreView imaging on needle concept. The objective lens is focused on the top surface of the compressed tissue that rests on the coring needle. Only the contacting parts (mechanical extension of the objective lens and the small specimen chamber) are shown in cross-section.

Supplementary video 2 **Video S2.** Video demonstration of CoreView imaging on needle workflow with animated computer-aided design drawings.

## Figures and Tables

**Table 1.** Biopsy thickness under compression

Percentage of compression	Original thickness	Target thickness	Marks turned

50	1.22 mm, 1.19 mm	0.61 mm, 0.60 mm	19.5, 19.2
40	1.12 mm, 1.22 mm	0.45 mm, 0.49 mm	21.4, 23.4
30	1.17 mm, 1.30 mm	0.35 mm, 0.39 mm	26.2, 29.1

**Table 2.** Nuclear intensity measurements from 10×porcine tissue images acquired with MUSE and from H and E brightfield

Nuclei and imaging modality	Minimum intensity	Maximum intensity	20% from baseline	80% from baseline	Pixel distance

MUSE 10 × Pig Nucleus 1	86.00	115.00	91.80	109.20	10.0
MUSE 10 × Pig Nucleus 2	86.50	111.00	91.50	109.20	11.0
MUSE 10 × Pig Nucleus 3	81.87	112.90	88.08	106.70	8.0
MUSE 10 × Pig Nucleus 4	97.00	124.09	102.40	118.70	9.0
MUSE 10 × Pig Nucleus 5	92.97	122.72	98.92	116.80	12.0
Average MUSE 10 × Pig					10.0
H&E 10 × Pig Nucleus 1	145.00	212.40	198.92	158.48	6.0
H&E 10 × Pig Nucleus 2	91.00	221.20	195.16	117.04	12.0
H&E 10 × Pig Nucleus 3	97.00	207.00	185.00	119.00	6.0
H&E 10 × Pig Nucleus 4	115.90	240.00	215.20	140.70	7.0
H&E 10 × Pig Nucleus 5	128.00	212.00	195.20	144.80	10.0
Average H and E 10 × Pig					8.2

Abbreviations: H&E: Hematoxylin and eosin; MUSE: Microscopy with ultraviolet surface excitation.

## Data Availability

Data used in this work are available from the corresponding author on reasonable request.
